# The role of TGFBI (βig-H3) in gastrointestinal tract tumorigenesis

**DOI:** 10.1186/s12943-015-0335-z

**Published:** 2015-03-24

**Authors:** Bing Han, Haolei Cai, Ying Chen, Bing Hu, Hongyu Luo, Yulian Wu, Jiangping Wu

**Affiliations:** Laboratory of Immunology and Cardiovascular Research, Centre hospitalier de l’Université de Montréal (CRCHUM), 900 Stain-Denis Street, Montreal, Quebec Canada; Department of Surgery, The Second Affiliated Hospital of Zhejiang University, 88 Jiefang Road, Hangzhou, China; Anatomic Pathology, AmeriPath Central Florida, 8150 Chancellor Dr, Orlando, FL USA; Nephrology Service, Research Centre, Centre hospitalier de l’Université de Montréal (CRCHUM), 900 Stain-Denis Street, Montreal, Quebec Canada

**Keywords:** βig-H3/TGFBI, Gastrointestinal tract tumors, Carcinogenesis, Transgenic mice, Gene knockout mice, Patient serum TGFBI levels

## Abstract

**Background:**

TGFβ-induced (TGFBI/βig-H3) is a protein inducible by TGFβ1 and secreted by many types of cells. It binds to collagen, forms part of the extracellular matrix (ECM), and interacts with integrins on cell surfaces. In this study, we investigated the role of TGFBI in tumorigenesis and the underlying mechanisms.

**Methods:**

Patient serum TGFBI levels were determined by ELISA. TGFBI transgenic and gene knockout mice and TGFBI-overexpressing liver cells were used for mechanistic studies.

**Results:**

We demonstrated that patients with cholangiocarcinomas, hepatic carcinomas or gastric carcinomas presented significantly elevated serum TGFBI levels, and the excess TGFBI was derived from the tumor masses. TGFBI overexpression in mice resulted in increased incidence of spontaneous tumors and N,N-diethylnitrosamine (DEN)-induced liver tumor nodules, compared to that in wild type (WT) mice, while TGFBI knockout mice were comparable to WT controls in these 2 aspects. TGFBI promoted the survival of Aml-12 liver cells with DNA damage after irradiation, and augmented their post-irradiation proliferation. It activated the FAK/AKT/AKT1S1/PRS6/EIF4EBP pathway, which is known to modulate cell survival and proliferation.

**Conclusions:**

Our data suggest that TGFBI functions as a promoter of certain gastrointestinal tract cancers. It provides a survival advantage to cells with DNA damage. Over a long time span, this advantage could translate into increased tumor risks.

## Background

TGFBI, also known as βig-H3, is a gene cloned from TGFβ1-stimulated A549 lung adenocarcinoma cells [1]. TGFBI protein has an N-terminal secretory signal (amino acids 1–23), 4 FAS1 homologous internal domains, and a cell attachment RGD domain at its C-terminus [2]. It is produced by many cell types [3,4].

Quite a few correlative studies have revealed conflicting roles of TGFBI as either tumor suppressor or promoter.

TGFBI down-regulation has been noted in various tumors. For example, TGFBI loss or down-regulation has been observed in human lung carcinoma samples by immunohistochemistry [5,6]. TGFBI mRNA is down-regulated in human and mouse mixed lineage leukemia [7]. TGFBI promoter hypermethylation, which suppresses TGFΒΙ expression, is found in ovarian carcinoma specimens [8]. TGFΒΙ promoter hypermethylation is also correlated with the metastatic phenotype of lung, prostate and ovarian carcinomas [8-11]. Lung carcinomas overexpressing TGFΒΙ show increased sensitivity to chemotherapy [12]. Decreased TGFΒΙ expression in ovarian carcinomas elicits Paclitaxel resistance [13]. TGFBI protein expression is reduced in breast carcinoma cells [14]. These findings suggest that TGFBI functions as a tumor suppressor.

On the other hand, TGFBI upregulation has also been reported to be associated with tumorigenesis. Immunohistochemistry has determined that human colon carcinomas manifest increased TGFBI expression [15]. Based on microarray analysis, TGFBI is among a number of genes up-regulated in human pancreatic carcinomas [16] and human squamous cell carcinomas [17]. mRNA expression and immunohistochemistry studies have revealed that TGFBI is one of the genes overexpressed abnormally in colon carcinomas and adenomas [18]. *In silico* analysis of a protein-protein interaction database has disclosed enhanced TGFBI expression by esophageal squamous cell carcinomas [19]. These findings suggests that TGFBI functions as a tumor promoter.

There are more causative evidences pointing to either the negative or positive involvement of TGFBI in tumorigenesis.

TGFBI is reported to have tumor suppressor-like functions. For example, TGFBI overexpression inhibits tumorigenesis and reduces the mobility of lung carcinoma cells [14,20]. TGFBI hampers neuroblastoma cell proliferation and invasion [21,22]. The C-terminal fragment of TGFBI induces human osteosarcoma and lung carcinoma cell apoptosis [23,24]. TGFBI knockdown promotes the proliferation of mesothelioma cells [25]. TGFBI evokes apoptosis of ovarian carcinoma cells [11]. Aged TGFBI gene knockout (KO) mice manifest increased tumor risks (37% versus 8.3% in wild type (WT)) mice at 13–20.5 months of age [26]. These KO mice also have a higher incidence of chemically-induced skin cancer (40% versus 5% in WT controls at 6 months after induction). Lung carcinoma cells transfected with a TGFBI-expressing construct fail to establish tumors in mice *in vivo* whereas empty vector-transfected cells do, indicating a suppressive effect of TGFBI on tumor formation [5].

However, multiple studies report a causative tumor-promoting function of TGFBI. TGFBI overexpression in colon carcinoma cells enhances their metastasis in mice *in vivo* [15]. Recombinant TGFBI promotes ovarian carcinoma cell mobility and invasiveness [11]. TGFBI interacts with integrin to promote hepatocellular carcinoma cell invasiveness, while TGFBI knockdown reduces it [27,28]. TGFBI knockdown also attenuates glioma cell invasiveness [29]. Recombinant TGFBI induces lung carcinoma A549 cell growth. Of course, invasiveness and mobility are not tumorigenesis, but both favor secondary tumor formation.

We conducted human and animal studies to assess the role of TGFBI in tumorigenesis in an attempt to better understand the reasons underlying conflicting observations on the pro- and anti-tumor effects of TGFBI.

## Results

### Elevated serum TGFBI levels in certain gastrointestinal tract cancer patients

We assessed serum TGFBI levels in 427 patients admitted to surgical wards of the Second Affiliated Hospital of Zhejiang University and diagnosed with various malignant tumors: cholangiocarcinomas (n = 22), pancreatic carcinomas (n = 68), hepatic carcinomas (n = 47), lung carcinomas (n = 80), gastric carcinomas (n = 36), mammary carcinomas (n = 32), colon carcinomas (n = 62), brain carcinomas (n = 16), malignant lymphomas (n = 13), esophageal carcinomas (n = 15), prostate carcinomas (n = 10), and osteosarcomas (n = 26). Two hundred and sixteen surgical patients, admitted to the same surgery wards during the same time period as were the malignant tumor patients but with non-tumor conditions, were considered as controls. Identical blood collection and sample preservation procedures were employed for the 2 cohorts.

**Figure 1:**
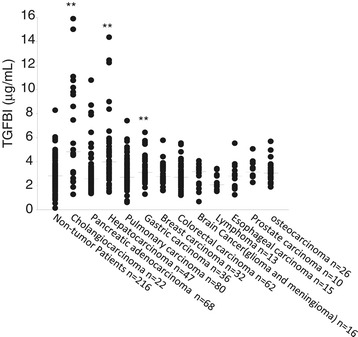
**Elevated serum TGFBI levels in patients with 3 types of gastrointestinal tract carcinomas.** Each patient serum sample was measured in duplicate for TGFBI levels with ELISA, and the mean of each sample was plotted as a dot, with tumor type and patient number indicated; the horizontal bars are group medians. TGFBI levels in patients of each tumor type were compared with those in control patients. Statistical significance is indicated by asterisks. **p < 0.01 (2-tailed Student’s *t* test).

Serum TGFBI levels non-malignant tumor patients and controls are reported in Figure 1 as scatter plots with horizontal bars indicating medians, to show their distribution in each tumor type. Compared to the non-tumor controls, cholangiocarcinoma, hepatocellular carcinoma, and gastric carcinoma patients presented significantly elevated serum TGFBI levels, with cholangiocarcinoma cases having the highest values. We pooled the data on all non-tumor patients and stratified them by age and gender, but neither of these 2 variables seemed to influence serum TGFBI levels (data not shown), suggesting that under the non-tumor condition, the age and gender do not influence the serum TGFBI levels. We also analysed TGFBI levels in cholagiocarcinoma, hepatocarcinoma, gastric carcinoma and pancreatic carcinoma patients for their association with clinical tumor stages, but no significant association was observed (data not shown).

**Figure 2:**
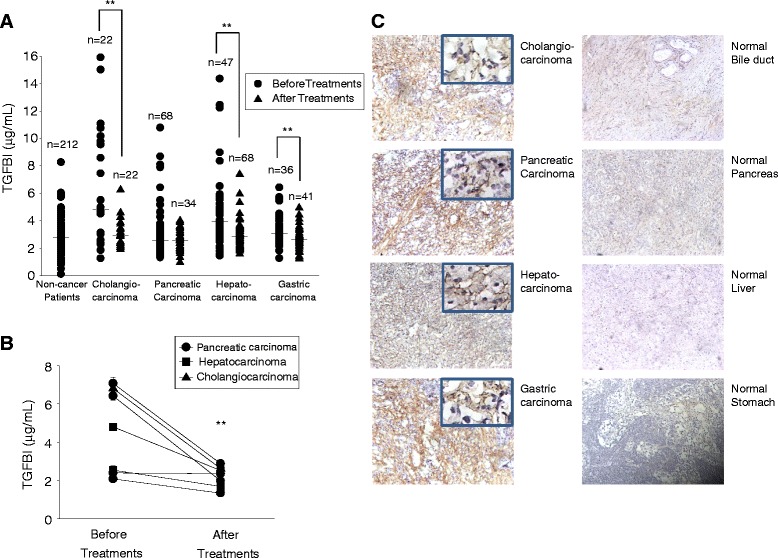
**Serum TGFBI levels in carcinoma patients before and after treatment. (A)** *Reduced serum TGFBI levels in carcinoma patients after treatment.* Serum TGFBI levels were measured in a cohort of patients with 4 types of gastrointestinal tract carcinomas. Serum TGFBI levels from a different cohort with the same 4 types of cancers were quantified after treatments (i.e., tumorectomy, chemotherapy, or irradiation). The results are scatter- plotted in *A*, with each dot or triangle representing mean serum TGFBI titre of a patient. The horizontal bars denote group medians. The numbers of patients in each category and carcinoma type are indicated. Serum TGFBI levels in non-tumor patients were included as controls. **: Statistically significant differences (*p* < 0.01) between pre- and post-treatment cohorts (2-tailed Student’s *t* test). **(B)** *Reduced serum TGFBI levels in a self-controlled cohort of carcinoma patients.* Serum TGFBI levels of 7 carcinoma patients (1 with cholangiocarcinoma, 3 with hepatocellular carcinomas, and 3 with pancreatic carcinomas) were measured before and after treatments (tumorectomy, chemotherapy and irradiation) and plotted. Levels after treatment were significantly lower than before treatment (**: *p* < 0.01; 2-tailed Student’s *t* test). **(C)** *Elevated TGFBI expression in certain gastrointestinal tract carcinomas according to immunohistochemistry.* Cryosections of human holagiocarcinoma, pancreatic carcinoma, hepatocarcinoma and gastric carcinoma (left column) were stained with anti-TGFBI Ab and TGFBI signals are revealed in brown. Insets with higher magnification show intracellular TGFBI staining. Normal tissues from these human organs were used as controls (right column).

We tested serum TGFBI levels of some tumor patients before and after treatment (tumorectomy, chemotherapy or irradiation). Because of logistic difficulties, the majority of patients tested before and after treatment were not the same ones. Serum TGFBI levels in patients before and after treatment are illustrated in scatter plots in Figure 2A. They were significantly lower in cholangiocarcinoma, hepatocellular carcinoma and gastric carcinoma patients after the treatment than before the treatment. Individual treatment subgroup (i.e., tumorectomy, chemotherapy or irradiation) did not present significant difference compared to the group without treatments, probably due to insufficient sample sizes. We managed to obtain sera from 7 tumor patients before and after the treatments as a self-controlled sub-cohort. Indeed, serum TGFBI levels dropped significantly after the treatment (Figure 2B). We assessed tumor sections from cholangiocarcinomas, pancreatic carcinomas, hepatocarcinomas and gastric carcinomas for TGFBI expression with immunohistochemistry. As shown in Figure 2C (left column), these tumor sections were rich in TGFBI signals, both intracellularly (see insets of higher magnification) and in ECM. The detection of TGFBI in ECM is expected as it binds to collagens after being secreted from the source cells. On the other hand, no obvious TGFBI signal was detectable in normal bile duct, pancreas live and stomach tissues (Figure 2C, right column).

Collectively, these data indicate that serum TGFBI levels in some gastrointestinal tract tumors are elevated, and the tumors are the major source of the elevated TGFBI in the blood. Further, the serum TGFBI levels are influenced by tumor treatment.

### TGFBI overexpression but not TGFBI KO increases spontaneous tumor incidence in aged mice

**Figure 3:**
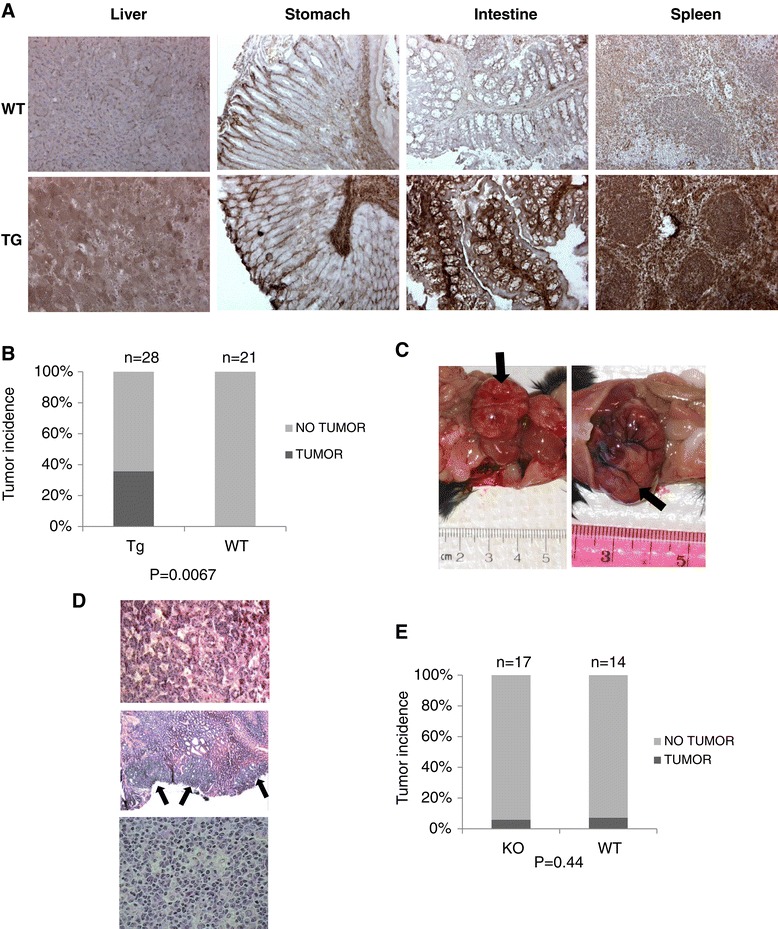
**Increased tumor incidence in aged TGFBI Tg but not TGFBI KO mice. (A)** *TGFBI overexpression in TGFBI Tg mice by immunohistochemistry.* Organs from WT and Tg mice were cryosectioned and stained with anti-TGFBI Ab and signals were revealed by Anti-mouse/rabbit Universal Immunohistochemistry Detection Kit (Proteintech Group). **(B)** *Tumor incidence in aged TGFBI Tg mice.* Tg and WT mice were kept under specific pathogen-free conditions. They were sacrificed and autopsied starting from age 16 months if one of the following conditions was met: 1) palpable lumps in abdomen; 2) loss of more than 20% body weight; 3) death from unknown causes; 4) reached age 22 months. Percentages of mice with tumor masses upon visual inspection are reported. The data were analyzed by 2-tailed Student’s *t* tests. *P*-value is indicated. **(C)** *Tumor masses in aged TGFBI Tg mice.* Arrows indicate tumor masses in 2 aged TGFBI Tg mice. **(D)** *Histology of tumors in aged TGFBI Tg mice.* Upper panel: hepatocellular carcinoma; middle panel: gastric adenoma, arrows indicate tumorous cells; lower panel: lymphoma-like tumor. **(E)** *Tumor incidence in aged TGFBI KO mice.* Tumor incidence in aged TGFBI KO and WT mice was determined as described in A. The data were analyzed by 2-tailed Student’s *t* test. *P*-value is indicated.

The clinical data demonstrated elevated serum TGFBI levels in patients with certain types of tumors. We wondered whether increased TGFBI levels caused tumorigenesis. To address this question, we assessed spontaneous tumor incidence in aged (>16-month-old) Tg mice with β-actin promoter-driven over-expression of TGFBI. Various organs of the Tg mice clearly overexpressed TGFBI, as this transgene was driven by an actin promoter (Figure 3A). Mice were sacrificed and/or autopsied starting from age 16 months if one of the following conditions was met: 1) palpable lumps in abdomen; 2) loss of more than 20% body weight; 3) death from unknown causes; 4) reached age 22 months. Spontaneous tumors were found in 10 out of 28 Tg mice (35.7%), but none was found in 21 WT controls (Figure 3B). At autopsy, these mice presented visceral tumor masses measuring 2 to 30 mm in greatest diameter (Figure 3C). Histologically, the tumor nodules or masses ranged from preneoplastic nodular hyperplasia to frank hepatocellular carcinomas in the liver (Figure 3D, upper panel), gastric intramucosal adenocarcinomas arising in adenomas (Figure 3D, middle panel), and malignant neoplasms histologically consistent with malignant lymphomas in the spleen (Figure 3D, lower panel, and data not shown). However, no WT littermates died or developed abdominal tumor masses during the same period. The difference was highly significant, indicating that TGFBI overexpression was tumorigenic.

Among KO mice, only 1 out of 17 (5.8%) developed visible tumor masses spontaneously between 16 and 22 months of age (Figure 3E), and was not statistically significantly different from WT littermates (1 out of 14; 7.1%).

### TGFBI overexpression but not TGFBI KO increases liver tumor incidence in mice fed DEN

**Figure 4:**
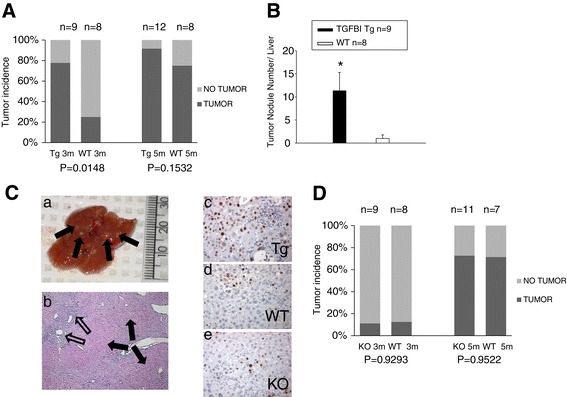
**TGFBI Tg but not TGFBI KO mice present increased incidence of carcinogen-induced liver pre-neoplasia. (A)** *Percentage of TGFBI Tg and WT mice with liver tumor nodules after DEN treatment.* TGFBI Tg mice and their WT littermates were treated with DEN in drinking water for 3 months (3 m) or 5 months (5 m) and then sacrificed. Percentages of mice with and without liver tumor nodules upon visual inspection are shown, and mouse number (n) per group indicated. *P*-values of Tg versus WT groups are presented (2-tailed Student’s *t* test). **(B)** *Number of tumor nodules on liver surface in Tg and WT mice treated with DEN.* TGFBI Tg mice and their WT littermates were treated with DEN in drinking water for 3 months. Visible tumor nodules on the liver surface were counted, means ± SD of nodules per liver reported, and mouse number (n) per group shown. The asterisk indicates significant statistical differences (*p* < 0.05, 2-tailed Student’s *t* test). **(C)** *Pre-neoplastic liver tumor nodules in mice treated with DEN in drinking water.* Arrows indicate multiple whitish tumor nodules on the liver surface from Tg mice on DEN-water drinking for 3 months (a). The liver was stained with hematoxylin-eosin; solid arrows indicate pre-neoplastic hepatocyte nodules; empty arrows point to cell infiltration around blood vessels (b). Representative liver sections from TGFBI Tg (c), WT (d) and TGFBI KO (e) mice, treated with DEN in drinking water for 3 months, were stained with anti-PCNA Ab. Signals appear in brown. **(D)** *Percentages of TGFBI KO and WT mice with liver tumor nodules after DEN treatment.* TGFBI KO mice and their WT littermates were treated with DEN in drinking water for 3 months (3 m) or 5 months (5 m), and the percentages of mice with liver tumor nodules are shown.

To corroborate observations based on spontaneous tumor incidence in aged mice, we tested the effect of TGFBI overexpression and KO in mice exposed to DEN. After 3-month DEN treatment, whitish tumor nodules of various sizes (normally less than 3 mm in diameter) developed on the liver surfaces of some mice. Seven out of 9 (77.8%) Tg mice but only 2 out 8 (25%) WT littermates developed tumor nodules in their livers (Figure 4A; left panel). The difference was statistically significant. Also, Tg mice presented significantly larger numbers of tumor nodules per liver than WT controls (Figure 4B).

Histologically, typical tumor nodules from Tg livers after 3 months of DEN treatment gave the impression of pre-neoplastic hepatic nodular hyperplasia (Figure 4C-a and C-b, solid arrows), along with chronic inflammation and bile ductule proliferation, most severe in portal and periportal areas (Figure 4C-b, empty arrows). In some Tg mice, proliferating bile ductules formed anastomosing and distorted tubules with epithelial dysplasia (data not shown). The degree of abnormal bile ductule proliferation and associated inflammation after DEN treatment was most severe in Tg mice and least severe in WT. While KO mice also demonstrated increased chronic inflammation and bile ductule proliferation compared to WT controls, their degree was less severe than that of Tg mice (data not shown). Proliferating cell nuclear antigen (PCNA) staining revealed that the nodules from Tg livers contained numerous actively-proliferating hepatocytes, while such nests were much fewer and smaller in WT and KO livers (Figure 4C-c, d, and e).

At 5 months of DEN treatment, most Tg (91.7%) and WT (75%) mice developed tumor nodules, and the difference was not statistically significant (Figure 4B, right panel), nor was the difference in numbers of tumor nodules per liver (data not shown).

In TGFBI KO mice and their WT littermates, DEN treatment caused no significant differences in tumor incidence after either 3 or 5 months, although, as expected, 5-month treatment induced greater tumor incidence than 3-month treatment (Figure 4D).

### TGFBI reduces liver cell apoptosis after DNA damage and promotes cell proliferation after irradiation

**Figure 5:**
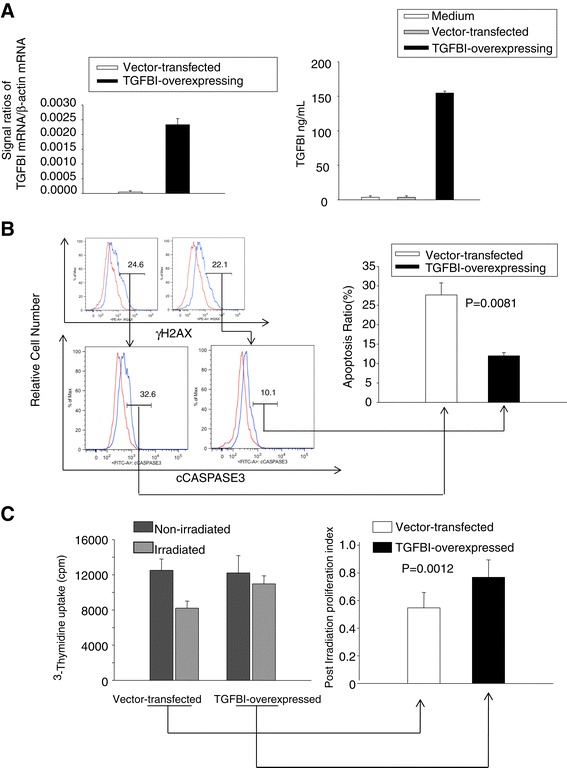
**TGFBI promotes liver cell survival and proliferation after irradiation. (A)** *Aml-12 liver cells overexpressed TGFBI after stable transfection with the TGFBI-expressing construct pCEP4-TGFBI.* TGFBI mRNA levels of transfected Aml-12 liver cells were measured by RT-qPCR. β-actin mRNA levels served as internal controls, and data are expressed as ratios of TGFBI versus β-actin signals (left panel). The supernatants of these cells were quantified by ELISA in duplicate for TGFBI protein levels; regular culture medium was included as an additional control (right panel). Means ± SD of representative experiments are shown. **(B)** *TGFBI promotes post-irradiation survival of liver cells carrying DNA damage.* TGFBI-overexpressing Aml-12 and control cells were irradiated at 20 Gy and cultured for 20 h. They were then stained with Abs against γH2AX and caspase-3, and analyzed by flow cytometry. The percentages of caspase-3-positive cells among γH2AX-positive cells are presented (left), and the percentages of 3 independent experiments are summarized in the bar graph (right), with *p*-value indicated (2-tailed Student’s *t* test). **(C)** *TGFBI promotes post-irradiation proliferation of liver cells.* TGFBI-overexpressing Aml-12 and control cells after irradiation were cultured in complete DMEM/F12 medium containing 10% FCS for 40 h. ^3^H-thymidine was added 16 h before the cells were harvested. Thymidine uptake of both irradiated and non-irradiated cells in a representative experiment is shown on the left. Post-irradiation proliferation indices of 3 independent experiments are summarized in a bar graph on the right with *p*-value indicated (2-tailed Student’s *t* test).

Aml-12 cells are mouse hepatocytes that are non-transformed but immortal due to transgenic human TGF expression. We stably transfected this cell line with a mouse TGFBI expression construct pCEP4-TGFBI. TGFBI overexpression by these cells at the mRNA level was proven by reverse transcription-quantitative polymerase chain reaction (RT-qPCR) (Figure 5A, left panel), and TGFBI protein overexpression was demonstrated by enzyme-linked immunosorbent assay (ELISA) of culture supernatants (Figure 5A, right panel).

Twenty h after 20-Gy irradiation, vector-transfected controls and TGFBI-overexpressing Aml-12 cells manifested similar levels of DNA damage according to H2AX staining (Figure 5B, upper left panel). We gated H2AX-positive cells and assessed their apoptosis by caspase-3 staining. As illustrated in the lower left panel of Figure 5B, TGFBI-expressing, H2AX-positive cells presented much less apoptosis (10.1%) than vector-transfected H2AX-positive cells (32.6%). Data from 3 independent experiments are summarized in Figure 5B (right panel). H2AX-positive, TGFBI-overexpressing Aml-12 liver cells manifested significantly lower apoptosis than their vector-transfected controls.

We next examined TGFBI-overexpressing Aml-12 cell proliferation after irradiation. Aml-12 cells readily form aggregates that are difficult to disperse. Consequently, it is technically challenging to precisely enumerate cell number and culture equal numbers of TGFBI-overexpressing and vector-transfected control cells. We, therefore, distributed the same type of cells in duplicate plates, one undergone irradiation, and the other cultured without irradiation. Cell proliferation was assessed by ^3^H-thymidine uptake, and uptake ratios between irradiated versus non-irradiated cells were considered as proliferation indices reflecting the impact of irradiation on proliferation, independent of precise cell counting. As illustrated in Figure 5C (left panel), irradiation inhibited the proliferation of control Aml-12 cells more strongly than that of TGFBI-overexpressing cells. The proliferation index of TGFBI-overexpressing cells in 3 independent experiments was significantly higher than that of control cells (Figure 5C, right panel), indicating that Aml-12 cells proliferate better post-irradiation in the presence of TGFBI.

### TGFBI activates the FAK/AKT/AKT1S1/mTOR pathway

**Figure 6:**
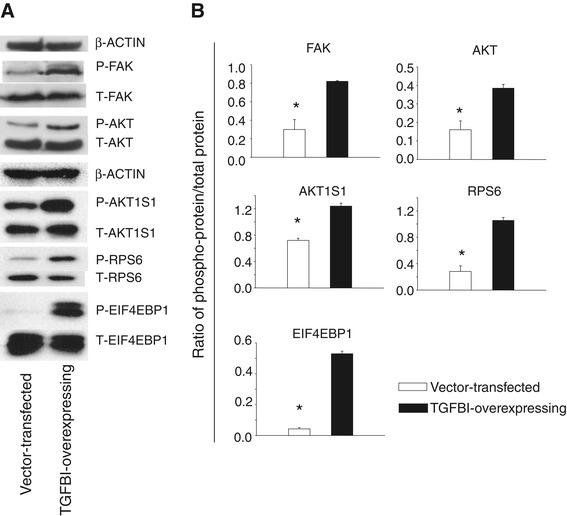
**TGFBI increases FAK, AKT AKT1S1, RPS6, and EIF4EBP1 phosphorylation in liver cells.** Aml-12 liver cells stably transfected with TGFBI-expressing vector (TGFBI-overexpressing) and empty vector (vector-transfected) were assessed for their FAK, AKT AKT1S1, RPS6, and EIF4EBP1 phosphorylation according to immunoblotting. Results of representative experiments are shown in **A**. Ratios of phosphorylated proteins versus total proteins in 3 independent experiments are summarized in **B**. with means ± SD indicated. Asterisks denote statistical significance (*p* < 0.05, 2-tailed Student’s *t* test).

TGFBI is known to bind integrins. In a previous study, we demonstrated that FAK, which is positioned upstream in the integrin signaling pathway, is activated in islets upon TGFBI stimulation [30]. We investigated TGFBI Tg islets by interrogating Full Moon BioSystem phospho-protein arrays containing Abs against 402 phosphorylated kinases, adaptor proteins, and transcription factors, looking for additional molecules involved in the TGFBI signaling pathway. A molecule known as AKT1S1 was confirmed to undergo increased phosphorylation [31]. AKT1S1 is a substrate of AKT, which is downstream of FAK, and it is also a component of the mTOR signaling pathway, which is critical in cell survival and proliferation [32]. We investigated whether this molecule and the signaling pathway it is associated with were also operative in liver cells. Immunoblotting demonstrated that AKT1S1 phosphorylation (Thr246) was significantly increased in TGFBI-overexpressing Aml-12 cells compared to vector-transfected control cells (Figure 6). Such up-regulation was accompanied by higher FAK(Tyr397) and AKT(S473) phosphorylation (Figure 6), reflecting enhanced upstream signaling strength responsible for AKT1S1 phosphorylation.

AKT1S1 phosphorylation allows it to dissociate from mTOR complex 1 (mTORC1) and renders mTOR active [33,34]. Indeed, we determined that 2 downstream mTOR molecules, i.e., RPS6 (S235/S236) and EIF4EBP1 (Thr37/Thr46), underwent increased phosphorylation in TGFBI-overexpressing Aml-12 cells (Figure 6), indicating mTOR signaling pathway activation due to TGFBI-triggered signaling.

## Discussion

Clinical and mechanistic results argue for TGFBI as both tumor suppressor and promoter. Convincing evidence supports both contentions [5-9,11-20,23-29]. Ween et al. [35] compiled an updated, comprehensive list of articles describing TGFBI as suppressor and promoter of different cancer types. The list gives the general impression that TGFBI is a promoter of gastrointestinal tract cancers, and functions as a tumor suppressor of other cancers. This impression is loosely corroborated by our data in that patients with 3 types of gastrointestinal tract carcinomas presented significantly higher serum TGFBI levels with excess TGFBI derived from the tumor masses, whereas they were not elevated in patients with malignant tumors of other origins, such as bone, prostate, breast, and hematopoietic cells (Figure 1). A few gastrointestinal tract carcinomas were the exception, e.g., colon carcinomas and pancreatic carcinomas. With that said, some patients with pancreatic carcinomas had high serum TGFBI levels, although TGFBI levels as a group were not significantly higher than in the controls (Figure 1). This has raised an intriguing possibility that the pro- and contra-tumor effects of TGFBI are tissue- and tumor type-dependent events.

Zhang et al. reported that aged TGFBI KO mice (13–20.5 months old) presented increased risks of spontaneous tumors: 37% in KO mice versus 8.3% in WT mice [26]. However, the TGFBI KO mice generated by us had no such phenotype. As depicted in Figure 3D, the incidence of spontaneous tumors in our aged KO and WT mice (16–22 months old) was 5.8% and 7.1% respectively, similar to that in WT controls described in Zhang’s study. In Zhang’s KO mice, exons 4–6 of the TGFBI gene was deleted, while in our KO mice, a more extensive deletion from exon 4 to 12 occurred. With that said, both Zhang et al. and we demonstrated convincingly that TGFBI in these 2 types of KO mice were deleted. So the degree of gene deletion is unlikely a reason for the observed discrepancy in the tumor phenotype. We have noticed that the majority (73.9%; 17 of 23) of tumors in Zhang’s investigation are lymphomas or due to lymphoproliferative disorder, raising the possibility that TGFBI KO mice are compromised in certain anti-virus immune responses, and Zhang’s colony had certain virus infections, increasing the incidence of lymphomas.

In our experiments, we demonstrated that high TGFBI expression is associated with 3 types of gastrointestinal carcinomas in patients and causes tumorigenesis in mice. We suspect that the basic mechanism underlying increased tumor incidence in Tg mice and sub-populations of TGFBI-overexpressing human cancer patients is that TGFBI promotes the survival of cells which have cancerous gene mutation. To prove our point, we tested whether TGFBI could promote survival of hepatocytes with DNA damage. Irradiation-induced DNA damage and DNA-damaged cells activate checkpoints at different phases of the cell cycle. Such cell cycle arrest has the benefit of giving cells a time window for DNA repair. Cells with severe DNA damage beyond repair will undergo senescence, or apoptosis, with or without exiting the checkpoints, whereas those with less damage will exit checkpoints and resume the normal cell cycle because of: 1) recovery, if the damage is repaired, or 2) adaptation, in which cycling cells still have DNA damage [36]. In adapted cells, DNA damage could be detected by the presence of H2AX [37]. We irradiated TGFBI-overexpressing Aml-12 liver cells, and found reduced apoptosis in H2AX-positive cells, compared to H2AX-positive control cells which had little TGFBI expression. Also, these TGFBI-expressing cells after irradiation had a higher proliferation index than control cells. Thus, it is quite possible that some adapted cells with DNA damage (hence mutations) in the presence of TGFBI have a survival and proliferation advantage over control cells. Of course, not all H2AX-positive cells will eventually survive in the presence of TGFBI, but in pathophysiological settings, the survival of even a tiny fraction of adapted cells over a long period of time will translate into increased incidence of tumors.

Although TGFBI-overexpressing Aml-12 cells showed reduced apoptosis after irradiation, the supernatants from these cells did not seem to protect vector-transfected Aml-12 cells from irradiation-induced apoptosis (data not shown). It is possible that the secreted TGFBI is quickly diluted in the culture medium and cannot reach to a sufficiently high concentration to protect nearby fraternal cells, while the concentration in the vicinity of the source cells is high enough to exert the protective autocrine effects against apoptosis. However, *in vivo* in tissues packed with cells, it is still possible for TGFBI from secreting source cells to protect other cells in the vicinity in a paracrine way, as there is less dilution under such circumstances. It is especially true as the secreted TGFBI will bind to collagens and accumulate in ECM. Such TGFBI-enriched ECM will likely provide nearby cells with a survival advantage.

We discovered that TGFBI augmented AKT1S1 (Thr246) phosphorylation. AKT1S1 is an AKT substrate that binds mTORC1 via Raptor and can inhibit mTORC1 autophosphorylation as well as its kinase activity [38]. AKT1S1 phosphorylation at Thr246 by AKT elicits its dissociation from mTORC1 [33], and releases its inhibitory effect on mTORC1 [34]. AKT1S1 can thus be considered as a “bridge” between signaling pathways involving AKT and mTORC1. mTORC1 has 2 kinase substrates. The first is p70S6K, whose phosphorylation and activation lead to RPS6 phosphorylation. RPS6 is a component of the complex ribosome machinery for protein synthesis. It has been proposed that ribosomes with the highest proportion of phosphorylated RPS6 become active and serve as part of polysomes [39]. EIF4EBP1, the second known mTORC1 substrate, is a repressor of protein translation [40]. Hypophosphorylated EIF4EBP1 interacts with EIF4E and prevents recruitment of the translation machinery to mRNA, thus inhibiting translation, whereas phosphorylated EIF4EBP1 enhances translation. The activation of FAK (which is upstream of AKT), AKT, RPS6 and EIF4EBP1n in liver cells by TGFBI was confirmed by Western blotting (Figure 6). Our data suggest that in liver cells, TGFBI could trigger signaling along the FAK/AKT/AKT1S1/mTOR pathway which, in turn, is known to promote cell survival [32] and reduce the death of cells with DNA damage or mutation, likely favouring tumorigenesis.

## Conclusions

In summary, we revealed that patients with cholangiocarcinomas, hepatocellular carcinomas or gastric carcinomas presented elevated serum TGFBI levels. TGFBI-overexpressing mice but not TGFBI KO mice showed increased incidence of spontaneous tumors and DEN-induced liver tumor nodules. Mechanistically, TGFBI promoted the survival and proliferation of liver cells with DNA damage, and activated the FAK/AKT/AKT1S1/PRS6/EIF4EBP pathway. Thus, TGFBI functions as a promoter of certain gastrointestinal tract cancers.

## Methods

### Patient cohorts

A cohort of 427 patients with confirmed diagnoses of various malignant tumors according to pathology was recruited to this study in the Department of Surgery, the Second Affiliated Hospital of Zhejiang University, Hangzhou, China. Blood was collected before treatment (tumorectomy, chemotherapy or irradiation) and, in some cases, after treatment. A cohort of 216 patients, admitted to the Department of Surgery for diseases other than malignant tumors, served as controls. These patients had no previous history of malignant tumors or symptoms, or laboratory findings indicating the existence of such tumors at the time of blood collection. Informed consent was obtained from patients before the donation of their blood samples for this study, which was performed in accordance with institutional ethics guidelines and was approved by the Second Affiliated Hospital Ethics Committee of Zhejiang University. Blood samples were rested at room temperature for 1–2 hours after being drawn from the patients. The samples were centrifuged at 1,400 g for 15 min at room temperature, and sera were collected and stored at −80°C until being assayed for TGFBI levels with ELISA. The serum TGFBI levels of patients of different groups tested were assessed by normal probability plots and were found to be of sufficient normal distribution for Student’s *t* tests.

### ELISA

Human and mouse TGFBI ELISA kits were purchased from R&D Systems (Minneapolis, MN). Human sera were diluted at 1:2000 with PBS for ELISA. Assays were undertaken as per the manufacturer’s instructions. All samples were tested in duplicate, and medians or means were computed for data presentation. Sensitivity was 6 pg/ml with human TGFBI ELISA, and 8 pg/ml with mouse TGFBI ELISA. Standard curves were constructed for each batch of ELISA using recombinant TGFBI. Inter-assay coefficient of variation was less than 5%.

### TGFBI transgenic (Tg) and KO mice

Tg mice with actin promoter-driven TGFBI overexpression and TGFBI KO mice were generated in our laboratory, and their TGFBI overexpression and KO at the mRNA and proteins levels were fully characterized in our previous publications [30,31]. All mice were housed under specific pathogen-free conditions and studied according to protocols approved by the institutional Animal Protection Committee of the CRCHUM.

### Immunohistochemistry

Immunohistochemistry for PCNA is described in our previous publication [31]. Briefly, paraffin-fixed sections of liver tissues were stained with mouse anti-PCNA mAb (Cell Signaling Technology, Danvers, MA; 1:100 dilution). Horse radish peroxidase-linked sheep anti-mouse IgG Ab (GE Healthcare, Little Chalfont, Buckinghamshire, UK; 1:200 dilution) served as the secondary Ab. PCNA signals were assessed by DAB substrate kit (BD Biosciences, Mississauga, Ontario, Canada). Details are also available in the standard immunohistochemistry protocol of Cell Signaling Technology.

For TGFBI protein expression in human cancers, normal tissues and TGFBI Tg and WT organs, frozen sections of tumors or tissues were stained with rabbit anti-TGFβi mAb (Proteintech Group, Chicago, IL; 1:50 dilution). TGFβi signals were revealed by Anti-mouse/rabbit Universal Immunohistochemistry Detection Kit (Proteintech Group). The sections were then counter-stained with hematoxylin.

### Induction of liver tumors with N,N-diethylnitrosamine (DEN)

Four- to 5-week-old male Tg, KO and WT mice in the C57BL/6 J background were given water containing 10 mg/l DEN (Sigma, St. Louis, MO) for 3 to 5 months before sacrifice. Liver tumor incidence was documented, as were tumor nodule number and size on liver surfaces.

### Plasmid construction and stable transfection

Full-length human TGFBI cDNA (MGC-1598; Open Biosystems/Thermo Scientific, Ottawa, Ontario, Canada) was cloned into mammalian cell expression vector pCEP4 (Invitrogen, Carlsbad, CA) between KpnI and NotI sites downstream of the CMV promoter. The resulting plasmid was named pCEP4-TGFBI. Mouse Aml-12 liver cells were transfected with pCEP4-TGFBI (2 μg/ml plasmid for 0.2 × 10^5^ Aml-12 cells/0.2 ml/well in 48-well plates) using X-treme GENE transfection reagent (Roche Diagnostics, Laval, Quebec, Canada) according to the manufacturer’s instructions. The cells were cultured in complete DMEM/F12 medium containing 10% fetal calf serum (FCS), 1× ITS Universal Culture Supplements (BD Biosciences), 1× MEM non-essential amino acids, 1 mM sodium pyruvate and 40 μg/l dexamethasone. Starting 48 h after transfection, 800 μg/ml hygromycin B (Invitrogen/Life Technologies) was present in the culture media for stable transfectant selection. TGFBI overexpression by stably transfected Aml-12 cells was confirmed by RT-qPCR and ELISA.

### RT-qPCR

Total RNA from Aml-12 liver cells was extracted with TRIzol (Invitrogen/Life Technologies). RNA was reverse-transcribed into cDNA with iScriptcDNA synthesis kits (Bio-Rad Laboratories, Hercules, CA). iQ SYBR Green Supermix PCR kits (Bio-Rad Laboratories) were employed for qPCR amplification of cDNA templates. The 5′ and 3′ primers were 5′-TTCCCTATTGTGACA GAGCCATGGT G-3′ and 5′-CACTGCATTCTAGTTGTGGTTTGTC C-3′, respectively. The PCR amplification program was as follows: 95°C, 3 min, 1 cycle; 95°C, 10 s, 59°C, 20 s, 72°C, 30 s, 45 cycles; with melting curves analyzed around 20–25 cycles. All samples were tested in duplicate. β-actin mRNA levels were considered as internal controls. In addition to melting curve analysis, qPCR products were verified by agarose gel electrophoresis for expected band sizes. The data are expressed as signal ratios of TGFBI mRNA/β-actin mRNA.

### Irradiation of Aml-12 cells

Aml-12 liver cells stably transfected with pCEP4-TGFBI or empty pCEP4 vector were irradiated at 20 Gy, and cultured in complete DMEM/F12 medium containing 10% FCS at 37°C for 20 h. They were assessed for DNA damage, apoptosis and proliferation.

### Flow cytometry

DNA damage and apoptosis of irradiated pCEP4-TGFBI- or empty pCEP4 vector-transfected Aml-12 cells were investigated by 2-color flow cytometry. They were first fixed and permeabilized using the eBioscience Fixation/Permeabilization Kit (eBioscience, San Diego, CA), and then stained with PE-conjugated rabbit anti-mouse phospho-histone H2AX (γH2AX) Ab and Alexa 488-conjugated rabbit anti-mouse cleaved caspase-3 Ab (Cell Signaling Technology).

### Post-irradiation proliferation of Aml-12 cells

Aml-12 cells were aliquoted with equal density into 2 sets of 96-well plates: one set was irradiated at 20 Gy, while the other was not. The cells were cultured for 48 h in complete DMEM/F12 medium containing 10% FCS. ^3^H-thymidine was added to culture 16 h before harvesting, as detailed previously [41]. Post-irradiation cell proliferation indices were calculated as follows:

Post-irradiation proliferation index = cpm of cells post-irradiation/cpm of cells without irradiation.

### Immunoblotting

Aml-12 liver cells stably transfected with pCEP4-TGFBI or empty pCEP4 vector were cultured overnight in serum-free DMEM/F12 medium containing 2% BSA, 1 × ITS Universal Culture Supplement, 1× MEM non-essential amino acids, 1 mM sodium pyruvate and 40 μg/l dexamethasone), and then lysed. Fifty μg of lysate proteins per lane were loaded for 10% SDS-PAGE. Proteins in the gels were transferred to polyvinylidene fluoride membranes after electrophoresis. The membranes were hybridized with rabbit Abs against total or phosphorylated mouse FAK (Tyr397), AKT (S473), AKT1S1 (Thr246), RPS6 (S235/S236), and EIF4EBP1 (Thr37/Thr46). All Abs were from Cell Signaling Technology and used at 1:1,000 dilution. Hybridization was performed according to Cell Signaling Technology instructions. Signals were detected with SuperSignal West Pico Chemiluminescent Substrate (Thermo Scientific).

### Data analysis

The data are expressed as means ± SD. Medians are indicated in some figures. Statistical significance (p < 0.05) was determined by 2-tailed Student’s *t* tests for data with normal distribution.

## Abbreviations

DEN: N,N-diethylnitrosamine
ELISA: Enzyme-linked immunosorbent assay
FCS: Fetal calf serum
γH2AX: Posphorylated histone H2AX
KO: Gene knockout
mTOR: Mammalian target of rapamicin
mTORC1: mTOR complex 1
PCNA: Proliferating cell nuclear antigen
RT-qPCR: Reverse transcription-quantitative polymerase chain reaction
Tg: Transgenic
TGFBI: Transforming growth factor beta-induced
WT: Wild type
